# ﻿A new species of *Sedum* (Crassulaceae) from eastern China based on morphological and molecular evidence

**DOI:** 10.3897/phytokeys.253.119922

**Published:** 2025-03-14

**Authors:** Jing-Min Dai, Yu Xiong, Pan Li, Yue-Liang Xu, Qiang Fan

**Affiliations:** 1 State Key Laboratory of Biocontrol and Guangdong Provincial Key Laboratory of Plant Stress Biology, School of Life Sciences, Sun Yat-sen University, Guangzhou 510275, China Sun Yat-sen University Guangzhou China; 2 Jiangxi Matoushan National Nature Reserve Administration, Fuzhou 335301, China Jiangxi Matoushan National Nature Reserve Administration Fuzhou China; 3 Laboratory of Systematic and Evolutionary Botany and Biodiversity, College of Life Sciences, Zhejiang University, Hangzhou 310058, China Zhejiang University Hangzhou China; 4 Zhejiang Museum of Natural History, Zhejiang, Hangzhou 310014, China Zhejiang Museum of Natural History Hangzhou China

**Keywords:** Eastern China, morphology, nr-ITS, *Sedum* sect. *Sedum*

## Abstract

*Sedumorientalichinense*, a new species of Crassulaceae from eastern China, is described and illustrated here. Phylogenetic analysis based on the internal transcribed spacer (ITS) region of nrDNA suggests that the new species belongs to S.sect.Sedum sensu Fu and Ohba (2001) in the “Flora of China”, and is sister to *S.makinoi* with high support values (BS = 100, PP = 1). The new species was previously always misidentified as *S.makinoi*, *S.emarginatum* or *S.baileyi*, due to its opposite leaves. *Sedumemarginatum* can be easily distinguished by its leaf blades with the apex emarginate in which it differs from the other three species. *Sedumorientalichinense* usually has 2-branched cymes, unlike *S.makinoi* which is 2- to 4-branched. The new species further differs from *S.makinoi* in its obovate to obovate-rhombic leaf blades (vs. obovate to obovate-spatulate in the latter) and has shorter stems (6–18 cm vs. 11–28 cm) with less internodes. It can also be easily distinguished from *S.baileyi* by its slender to sub-woody suberect stems (vs. slender and erect stems) and larger plant height (6–18 cm vs. 3–7 cm).

## ﻿Introduction

*Sedum* L. is the largest genus of the family Crassulaceae, including approximately 470 species of predominantly succulent plants (Thiede and Eggli 2007). The genus is predominantly distributed in temperate and subtropical areas of the Northern Hemisphere with diversity centers in the Mediterranean Region, Central America, the Himalayas, and East Asia (Stephenson 1994; Thiede and Eggli 2007). The succulent leaves and stems of *Sedum* allow them to store water and tolerate dry conditions, making them adapted to harsh environments such as deserts, cliffs, rocky or sandy areas, and meadows (Thiede and Eggli 2007).

According to Fu and Ohba (2001) in the “Flora of China”, the genus has three sections in the area, S.sect.Sedum, S.sect.Oreades (Fröd.) K.T. Fu, and S.sect.Filipes (Fröd.) S.H. Fu. Section Sedum can be separated from sections *Oreades* and *Filipes* by adaxially gibbous carpels and follicles, while sect. Oreades differs from sect. Filipes in having a spurred (vs. spurless) leaf base and petals that are mainly yellow (vs. white).

During the past 20 years, 18 new species have been published from China, including *S.hoi* X.F. Jin & B.Y. Ding (Wang et al. 2005), *S.fanjingshanense* C.D. Yang et X.Y. Wang (Yang et al. 2012), *S.kuntsunianum* X.F. Jin, S.H. Jin & B.Y. Ding (Jin et al. 2013), *S.plumbizincicola* X.H. Guo et S.B. Zhou ex L.H. Wu (Wu et al. 2012), *S.tarokoense* H.W. Lin & J.C. Wang (Lu et al. 2013), *S.spiralifolium* D.Q. Wang, D.M. Xie & L.Q. Huang (Xie et al. 2014), *S.peltatum* M.L. Chen et X.H. Cao (Chen et al. 2017), *S.ichangensis* Y.B. Wang (Wang and Xiong et al. 2019), *S.kwanwuense* H.W. Lin, J.C. Wang & C.T. Lu (Lu et al. 2019), *S.lipingense* R.B. Zhang, D. Tan & R.X. Wei (Zhang et al. 2019), *S.taiwanalpinum* H.W. Lin, J.C. Wang & C.T. Lu (Lu et al. 2019), *S.nanlingense* Yan Liu & C.Y. Zou (Zou et al. 2020), *S.danxiacola* S.Y. Meng & B. Chen (Meng and Chen et al. 2023), *S.jinglanii* Yan S. Huang & Q. Fan (Huang et al. 2023), *S.matsuense* C.T. Lu & W.Y. Wang (Lu and Wang et al. 2023), *S.yangjifengensis* B. Chen & Z.W. Zhu (Zhu et al. 2023), *S.fluviale* B. Chen & Z.W. Zhu (Zhu et al. 2024) and *S.xunvense* Y.L. Xu & P. Li (Chai et al. 2024). According to the Flora of China records by Fu and Ohba (2001), China originally documented 121 *Sedum* species with 91 endemics. The listed additional records (as of 2024) have updated these figures to 139 recognized species, including 109 endemic taxa.

During extensive field investigations in eastern China (Jiangxi, Zhejiang, and Anhui Provinces), we found a unique *Sedum* species growing on a rocky slope with opposite leaves, and usually 2-branched cymes. Through comprehensive literature studies, morphological comparison with related species and molecular analysis, we confirmed that it was a new species and provide a detailed description and illustration of it here.

## ﻿Methods

We conducted detailed field investigations and observations of the putative new species during its flowering and fruiting stages, and cultivated some plants in the laboratory for the study of its morphology. Morphological data were obtained by measurements based on abundant living samples from three different localities: Matoushan of Jiangxi Province (MTS), and Baizhangji (BZJ) and Daciyan (DCY) of Zhejiang Province (Fig. 5). Morphologically related species were collected from Lushan Mountain (LS) of Jiangxi Province (*S.baileyi*), Huangshan Mountain (HS) of Anhui Province and Yuyao (YY) of Zhejiang Province (both *S.emarginatum*). Additionally, numerous digital specimens were studied from online resources: Chinese Virtual Herbarium CVH (https://www.cvh.ac.cn/); Vascular Plants Herbarium of the Komarov Botanical Institute RAS—Herbarium LE (https://en.herbariumle.ru/); Global Biodiversity Information Facility GBIF (https://www.gbif.org/); Kagoshima University MuseumKAG (https://dbs.kaum.kagoshima-u.ac.jp/musedb/s_plant/s_plant.php); and Kagoshima University MuseumTSN (https://db.kahaku.go.jp/webmuseum/search?cls=col_b1_01) to obtain morphological data for related species. Data of *S.makinoi* were obtained from 33 specimens, and data of the putative new species were from 33 individuals. We measured the plant height and selected three mature leaf blades from each individual to measure the length and width of the leaf blade and calculated the average value. Since the Shapiro-Wilk test results showed that the three data sets did not follow a normal distribution, the two-sample Mann-Whitney U test was performed using SPSS 27.0.1.0 (2020). Type specimens were collected in the Matoushan National Nature Reserve, Jiangxi Province, China, and were deposited in the Herbarium of Sun Yat-sen University (SYS!).

The putative new species was sampled at three localities: Matoushan of Jiangxi Province (MTS, 3 individuals, *Xiong Y. 23062901*), and Daciyan (DCY, 1 individual, *Dai J.M. 24040701*) and Baizhangji (BZJ, 1 individual, *Dai J.M. 24040302*) of Zhejiang Province. Fresh leaves of the five individuals were collected and stored with silica gel in zip-lock plastic bags until use. Total DNA was extracted using the modified CTAB method (Doyle and Doyle 1987). For the amplification of the partial internal transcribed spacer 1, the 5.8S ribosomal RNA gene and the partial internal transcribed spacer 2 region we used the primers ITS1 and ITS4 (White et al. 1990). PCR amplifications were performed following Huang et al. (2021).

In order to explore the phylogenetic position of the putative new *Sedum* species, we downloaded ITS sequences of 56 accessions representing 46 *Sedum* taxa and three outgroup species from the Genbank database at the National Center for Biotechnology Information (NCBI) (Suppl. material 1). The selection of the three outgroup species (*Greenoviaaizoon*, *Aeoniumlancerottense*, *A.viscatum*) followed Huang et al. (2023). The sequences were aligned using MAFFT v. 7.520 (Katoh and Standley 2013). Based on Maximum likelihood (ML) and Bayesian Inference (BI), phylogenetic reconstructions were run by IQ-TREE v. 2.0.3 (Nguyen et al. 2015, Minh et al. 2020) with 2000 bootstraps (Hoang et al. 2018), and MrBayes version 3.1.2 (Huelsenbeck and Ronquist 2001), respectively. BI analysis used random starting trees and four Markov Chain Monte Carlo (MCMC) simulations were run simultaneously and sampled every 1000 generations for 30 million generations. Bayesian posterior probabilities (PP) were calculated as the majority consensus of all sampled trees with the first 25% discarded as burn-in. Figtree v. 1.4.3 (Rambaut 2016) was employed to visualize the tree.

## ﻿Result and discussion

The ITS sequences were aligned to a length of 732 bps, and 349 positions were parsimony-informative. The best-fit nucleotide substitution model was determined as SYM+I+G4 based on the Bayesian Information Criterion (BIC). The 5 samples of the new species had 5 variable sites within itself. There were 18 variable sites between the new species and *S.makinoi*, and 56 variable sites between the new species and *S.baileyi* and *S.emarginatum*, respectively.

**Figure 1.**
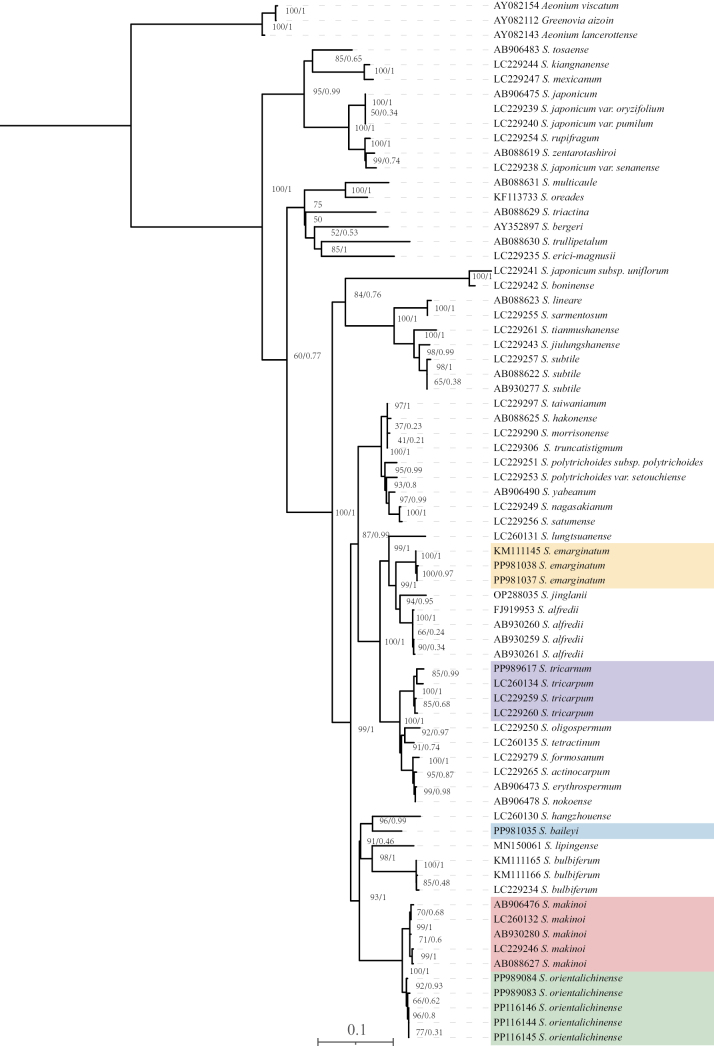
Maximum Likelihood (ML) tree based on ITS sequences of *Sedumorientalichinense* and related species. Bootstrap values of the ML and BI posterior probabilities are shown at the nodes. Three outgroups were used: *Greenoviaaizoon*, *Aeoniumlancerottense*, and *A.viscatum*. The new species is shaded in green. *Sedummakinoi*, *S.emarginatum*, and *S.baileyi* are shaded in red, yellow and blue, and *S.tricarpum* in purple, respectively.

According to the topology produced (Fig. 1), the five accessions of the new species formed a clade well supported by ML (BS = 92%, PP = 0.93). It was resolved as a well-supported sister (BS = 100%, PP = 1) to a robust (BS = 99%, PP = 1) *S.makinoi* clade. As shown in the ML phylogenetic tree, the morphologically similar species *S.baileyi* and particularly *S.emarginatum* placed (widely) separate the new species from *S.makinoi*.

Morphologically, the putative new species is similar to *S.makinoi*, but can be distinguished by its usually 2-branched (rarely 3-branched) cymes (vs. 2–4-branched in the latter), the shape of the leaf blades (obovate to obovate-rhombic vs. obovate to obovate-spatulate), and its plant height (6–18 cm vs. 11–28 cm) (Table 1). Although the new species was previously often misidentified as *S.baileyi* due to its opposite leaves, it can be easily distinguished from the latter by its slender to sub-woody stems (vs. slender stems), usually larger plant height (6–18 cm vs. 3–7 cm) (Table 1). Additionally, the Mann-Whitney U test results showed that the median plant height of *S.makinoi* was 16 (12.3, 20) and that of *S.orientalichinense* was 9.5 (7.875, 16.85), with statistically significant differences between the two groups (Z = 3.633, p = 0.01). The same conclusion can be drawn from the calculation results of leaf width (Table 2). These morphological differences support classifying *S.orientalichinense* as a new species. *Sedummakinoi* was previously given as distributed in Zhejiang and Anhui Province in China as well as Japan (Fu and Fu 1984; Fu and Ohba 2001). Supporting specimens were sampled from Huangshan, Anhui Province (Production practice team of Department of Biology of FDU 0338, PE00914380), and Siming Mountain, Zhejiang Province (*Siming Mountain 0577*, PE00914382, PE00914383). Based on morphology and phylogeny, the specimen collected from Huangshan is *Sedumtricarpum* Makino (Figs 1, 7). Xia and Liu et al. (2011) first recorded *S.tricarpum* from Anhui Province, China. This finding supports that the specimen (Production practice team of Department of Biology of FDU 0338, PE00914380, CVH) is misidentified as *S.makinoi* due to its occasionally opposite leaves. We could not find specimens of *S.makinoi* in Siming Mountain, Zhejiang, as was indicated by the specimens (*Siming Mountain 0577*, PE00914382, PE00914383). We believe the specimen from Siming Mountain and the new species are conspecific based on careful comparison of the morphology, such as 2-branched cymes and short stems (6.5–14.5 cm).

**Table 1.** Morphological comparisons between *Sedumorientalichinense*, *S.baileyi*, *S.makinoi* and *S.emarginatum*.

Character*	Species			
	*S.orientalichinense*	*S.makinoi*	*S.baileyi*	*S.emarginatum*
Plant height	6–18 cm	11–28 cm	3–7 cm	10–27 cm
Flowering stems	Suberect, slender to sub-woody, 4–8 internodes	Erect to suberect, slender to sub-woody, 8–12 internodes	Erect, slender	Suberect, slender
Phyllotaxy	Opposite	Opposite	Opposite	Opposite
Leaf blade	Obovate to obovate-rhombic, base tapered and shortly spurred, apex obtuse	Obovate to obovate-spatulate, base cuneate and shortly spurred, apex subacute	Obovate to obovate-rhombic, base tapered and shortly spurred, apex subacute	Spatulate-obovate to broadly obovate, base attenuate and shortly spurred, apex rounded and emarginate
Leaf length × width	1.3–2.7 × 0.6–2.4 cm	1.0–2.5 × 0.6–0.8 cm	Ca. 1.5 × 0.6 cm	1.5–2 × 0.5–1 cm
Inflorescence	Cymes usually 2-branched, rarely 3-branched	Cymes 2–4-branched	Cymes 1–2-branched, few flowered	Cymes usually 3-branched
Inflorescence diam.	3–10 cm	3–15 cm	1.5–3.5 cm	3–6 cm
Sepal shape	Spatulate-obelliptic, base shortly spurred, apex obtuse	Linear-spatulate, base shortly spurred, apex obtuse	Oblong-linear, basal spur broad and obtuse	Lanceolate to narrowly oblong, base shortly spurred, apex obtuse
Sepal length × width	2–4 × 1–1.5 mm	3–4 × 0.7–1 mm	1.5–2 × ca. 1 mm	2–5 × 0.7–2 mm
Petal length × width	3–5 × ca. 1 mm	4–6 × 1–2 mm	4–5 × ca. 1.5 mm	6–8 × 1.5–2 mm
Stamen size	Antesepalous ones subequaling petals; antepetalous ones shorter than petals	Antesepalous ones subequaling petals; antepetalous ones shorter than petals	Shorter than petals	Shorter than petals
Nectar scales	Broadly cuneate to sub-quadrangular	Oblong-spatulate	Oblong-spatulate	Oblong to broadly cuneate
Flowering	June–July	June–July	April	May–June
Fruiting	July	July	July	July
Distribution	China (Jiangxi, Zhejiang)	Japan	China (Guangdong, Guangxi, Hunan, Jiangxi)	China (Anhui, Gansu, Hubei, Hunan, Jiangsu, Jiangxi, Shaanxi, Sichuan, Yunnan, Zhejiang)

Abbrevistions: *Data of *S.orientalichinense* were from 33 individuals, data of *S.makinoi* were from 33 specimens and the “Flora of China”, data of *S.baileyi* and *S.emarginatum* were from the “Flora of China” (Fu and Ohba 2001).

**Table 2.** Quantitative characteristics and significance difference analysis of the species *Sedummakinoi* and *Sedumorientalichinense*.

Value	Species	M (P25, P75)	Mann-Whitney U test	
			Z	P
ph (cm)	*S.makinoi*	16 (12.3, 20)	3.633	0.001*
	*S.orientalichinense*	9.5 (7.875, 16.85)		
ll (cm)	*S.makinoi*	1.5 (1.3,1.9)	1.745	0.081
	*S.orientalichinense*	1.7 (1.588,1.863)		
lw (cm)	*S.makinoi*	0.55 (0.47,0.7)	3.821	0.001*
	*S.orientalichinense*	0.83 (0.67,0.95)		
ll/lw	*S.makinoi*	2.6 (2.16, 3)	3.056	0.002*
	*S.orientalichinense*	2.07 (1.8, 1.46)		

Abbreviations: ph = plant height; ll = leaf length; lw = leaf width. Independent-Sample Mann-Whitney Test was used, pl and lw representing significant differences at the 0.5% nominal level. M(P25, P75) means Median and Interquartile range, * means P < 0.05.

Although *S.makinoi* had not been found on Siming Mountain, as was indicated by the specimen cited above, some specimens morphologically closely related to *S.makinoi* were collected from other sites in Zhejiang Province (*Dai J.M. 24040302*, *Dai J.M. 24040701*). These specimens clustered together with *S.orientalichinense* (Fig. 1) and shared a similar morphology with it. This finding may suggest that the species previously misidentified as *S.makinoi* in China was actually *S.orientalichinense*, and that *S.makinoi* is absent from China and restricted in its distribution to Japan.

### ﻿Taxonomic treatment

#### ﻿ Sedum orientalichinense

Taxon classificationPlantaeSaxifragalesCrassulaceae

Q.Fan & P.Li sp. nov.

E3E7F0C8-DBAB-5740-86AD-5DF589D2928D

urn:lsid:ipni.org:names:77358282-1

Figs 2–6

##### Type.

China • Jiangxi Province, Fuzhou City, Mount Matoushan, Baishakeng, on rocky cliff, 27.77°N, 117.23°E, 424 m a.s.l., 29 June 2023, *Xiong Y. 23062901* (holotype: SYS00236991).

##### Diagnosis.

The new species differs from *S.makinoi* in its usually 2-branched cymes. *S.makinoi* also has longer, more erect stems with more internodes (8–12 vs. 4–8), resulting in denser foliage, whereas the new species has fewer leaves. Key differences include the shape and width of the leaf blades of *S.orientalichinense* and *S.makinoi* (obovate to obovate-rhombic vs. obovate to obovate-spatulate; 0.6–2.4 cm vs. 0.6–0.8 cm), shorter stems (6–18 cm vs. 11–28 cm), and a usually smaller inflorescence diameter (3–10 cm vs. 3–15 cm). The new species also has distinct sepals (spatulate-obelliptic vs. linear-spatulate) and nectar scales (broadly cuneate to sub-quadrangular vs. oblong-spatulate). Additionally, our research indicates that *S.makinoi* is endemic to Japan, which helps to distinguish the two species geographically. The new species can be distinguished from *S.baileyi* by its robust, slender to sub-woody stems (vs. slender stems) and greater height (6–18 cm vs. 3–7 cm) (Table 1). Lastly, *S.emarginatum* is easily identified by its emarginate leaf apex, a feature not found in the other three species (Table 1).

**Figure 2.**
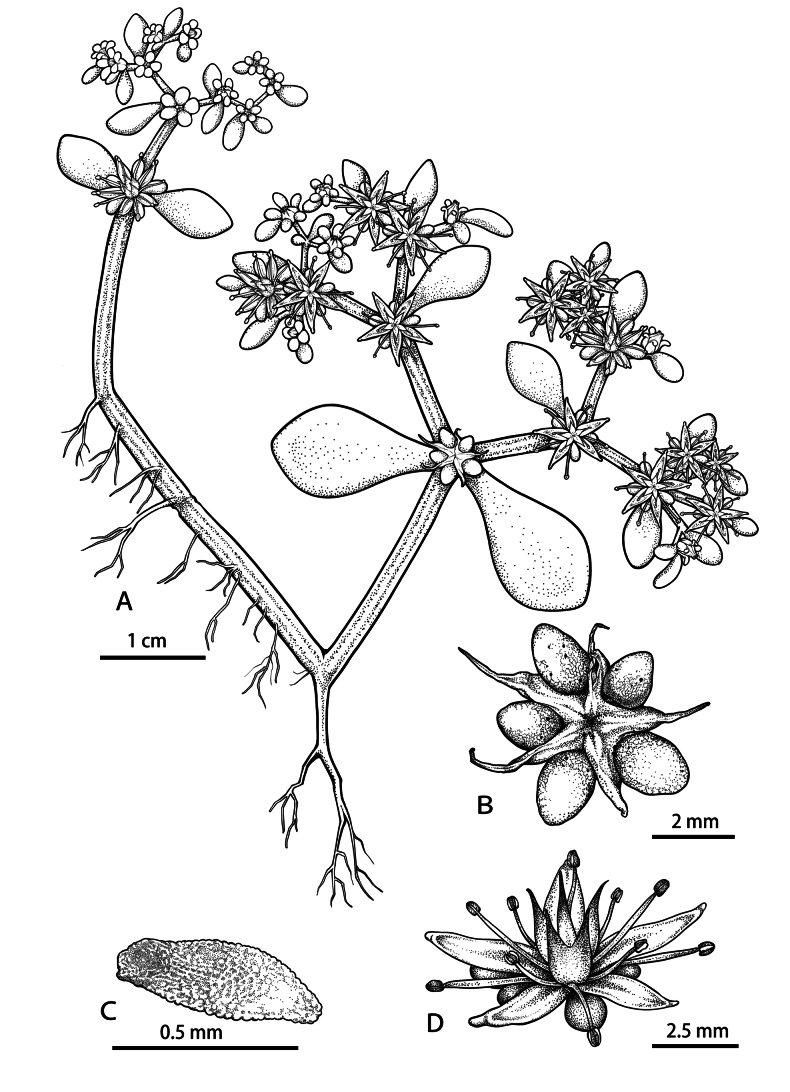
*Sedumorientalichinense***A** habit: Flowering stem with inflorescences **B** unripe follicles with sepals **C** seed **D** flower.

##### Description.

Perennial herbs, fleshy, glabrous, roots fibrous, stems slender to sub-woody, basally prostrate and rooting at nodes, apically erect, usually branched, rarely single, flowering stems sub-erect, with 4–8 internodes, usually 2-branched, rarely 3-branched, 6–18 cm high. Leaves opposite, glabrous, pseudopetiolate; leaf blade obovate to obovate-rhombic, margin entire, 1.3–2.7 × 0.6–2.4 cm, base tapered and shortly spurred, apex obtuse. Cymes usually 2-branched, rarely 3-branched, many flowered, 3–10 cm in diam. Bracts resembling stem leaves but obovate and smaller, 0.3–2.1 × 0.1–1.2 cm. Flowers sessile, equally 5-merous. Sepals 5, usually equal, rarely subequal, spatulate-obelliptic, 2–4 × 1–1.5 mm, base shortly spurred, apex obtuse. Petals yellow, base connate for ca. 0.3 mm, 3–5 × ca. 1 mm, lanceolate, apex acuminate, hooded. Stamens 10, in 2 whorls, both antesepalous ones and antepetalous ones shorter than petals, the antepetalous stamens fused at base for about 0.5 mm with the petal base, and the antepetalous ones slightly shorter the than antesepalous ones (1.3–1.4 vs. 0.9–1 cm). Nectar scales broadly cuneate to sub-quadrangular, ca. 0.5 mm long. Carpels 5, lanceolate, 4–5 mm long, connate at base for ca. 1 mm, apically usually divergent, sometimes closely connivent. Follicles obliquely divergent, stellate, many seeded, placentation marginal. Styles ca. 1 mm long. Seeds sub-ovoid, mammillate, brown when mature, 0.3–0.5 mm long.

**Figure 3.**
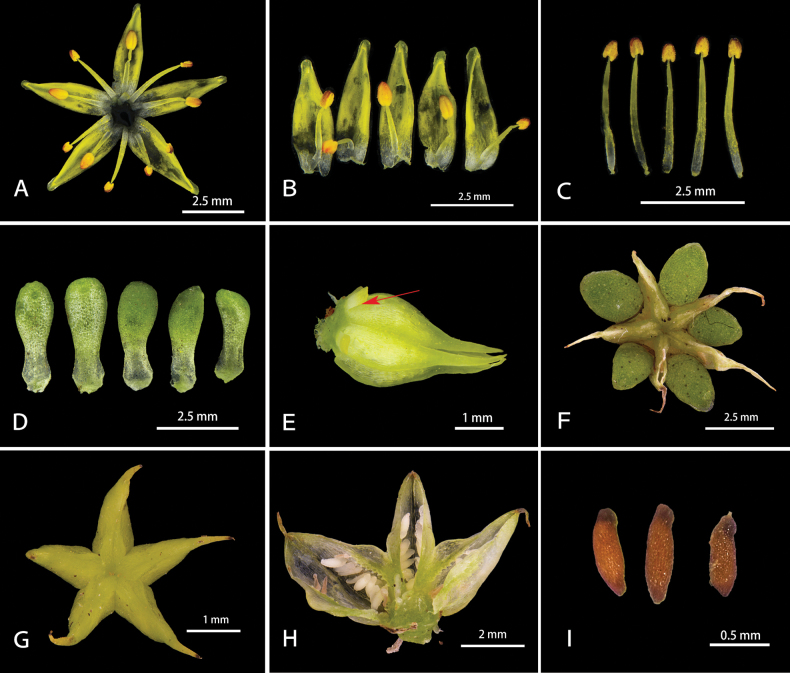
*Sedumorientalichinense***A** flower **B** petals and epipetalous stamens **C** episepalous stamens **D** sepals **E** carpels with nectar scale (marked with red arrow) **F** unripe follicles with sepals **G** unripe follicles **H** opened unripe follicles **I** seeds (photographs taken from plants cultivated at Sun Yat-sen University, Guangzhou Province, with **A–E** in June 2023, **F–H** in July 2023, **I** in July 2024, from the collection *Xiong Y. 23062901*).

##### Phenology.

Flowering from June to July, fruiting in July.

**Figure 4.**
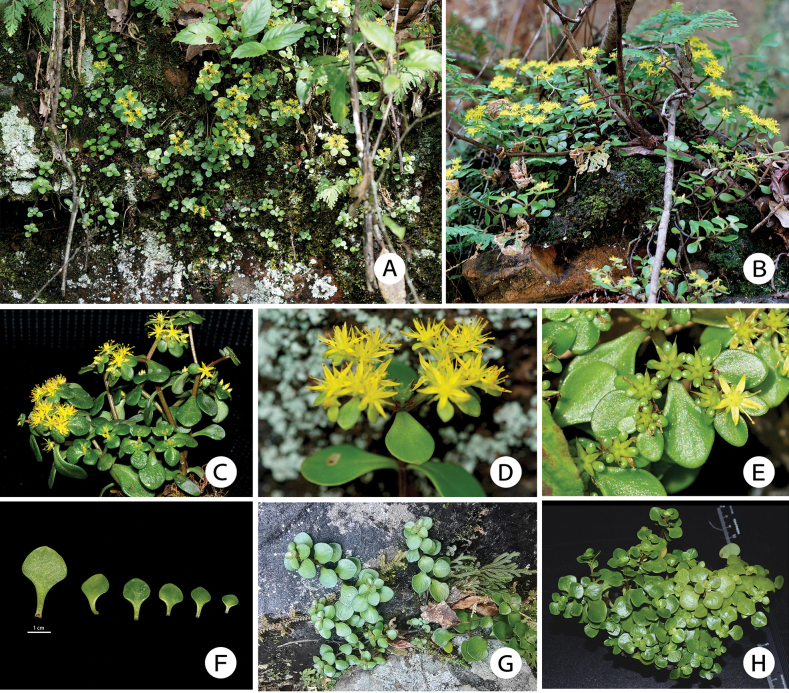
*Sedumorientalichinense***A** habitat **B, C** habit showing the sub-erect, 2-branched stems **D** side view of a cyme **E** cyme with unripe follicles **F** leaves from a single stem of *S.orientalichinense***G** sterile stems in the wild **H** vegetative growth of *S.orientalichinense* under artificial light in cultivation (**A, B, D, E, G** were photographed by Yu Xiong in Matoushan, Jiangxi Province in June 2023; **C, F, H** were photographed by Jing-Min Dai cultivated at Sun Yat-sen University, Guangzhou Province, with **F, H** in December 2023, and **C** in July 2024).

##### Etymology.

The specific epithet refers to the distribution area of the species.

##### Vernacular name.

We propose a Chinese name, Huá dōng Jǐng Tiān (华东景天).

**Figure 5.**
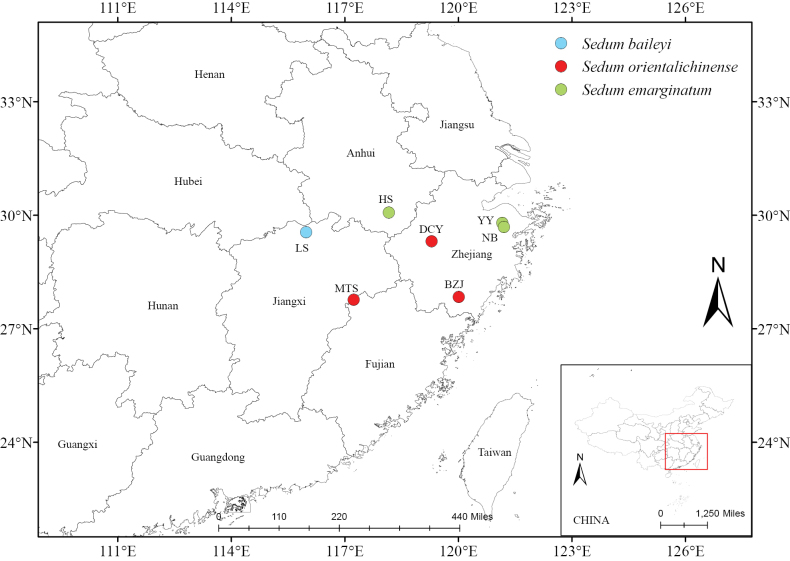
Distribution of *Sedumorientalichinense* and sampling sites of morphologically similar species. *Sedumorientalichinense* was sampled at its three known localities Matoushan (MTS, Jiangxi Province), Baizhangji (BZJ, Zhejiang Province), and Daciyan (DCY, Zhejiang Province). *S.baileyi* was sampled at the type locality Lushan Mountain (LS), Jiangxi Province. *S.emarginatum* was sampled at three sites: Yuyao (YY) and Ningbo (NB) in Zhejiang Province, and Huangshan (HS) in Anhui Province.

##### Distribution and habitat.

The new species is distributed in eastern China in Jiangxi and Zhejiang, provinces. It grows in rocky crevices and soil slopes in valleys at altitudes of 300–600 m a.s.l.

##### IUCN conservation status.

Due to its wider distribution and numerous individuals at each of the three investigation sites, *S.orientalichinense* should be considered as least concern (LC) (IUCN Standards and Petitions Subcommittee 2022).

**Figure 6.**
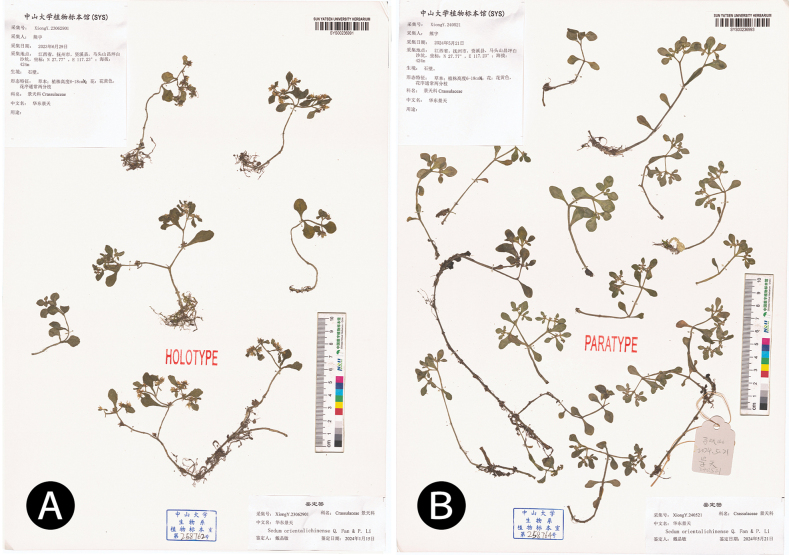
Type specimens of *Sedumorientalichinense***A** the holotype (*Xiong Y. 23062901* [SYS00236991]) **B** a paratype (*Xiong Y. 240521* [SYS00236993]).

**Figure 7.**
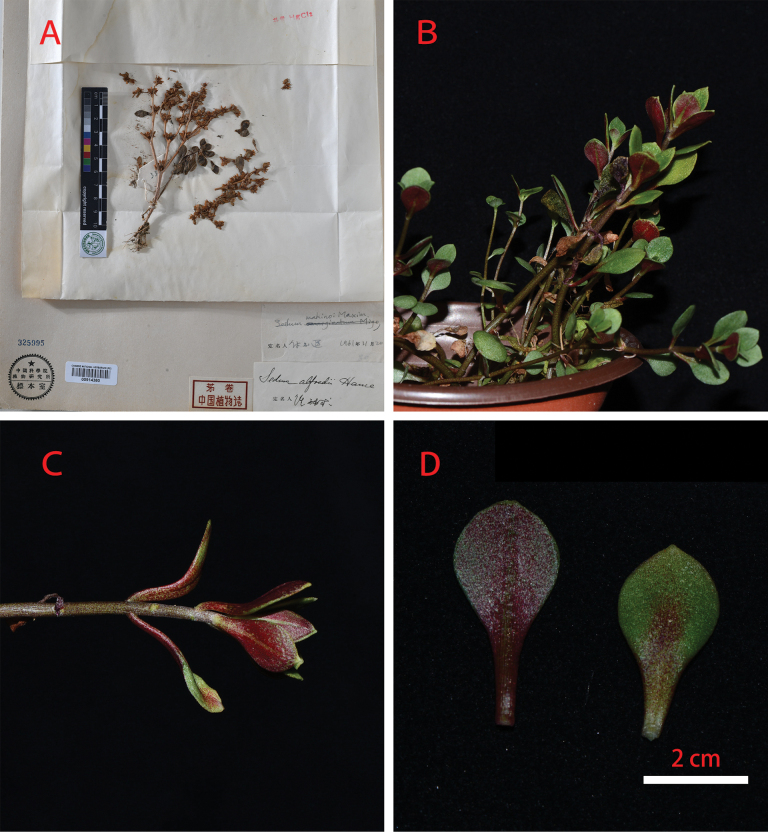
*Sedumtricarpum* from Huangshan **A** specimen from the Herbarium of the Institute of Botany, Chinese Academy of Sciences (Production practice team of Department of Biology of FDU 0338, PE00914380, Beijing, photographed by Bin-bin Liu) **B–D** living samples of *S.tricarpum* from Huangshan, Anhui province **B** habit **C** the leaves may occasionally be opposite which leads to misidentifications **D** leaf blade shape. (3 July 2024, photographed and collected by Jing-Min Dai, **B–D** were from a cultivated individual, *Dai J.M.2403221*).

##### Additional specimens examined.

***Sedumorientalichinense* (paratypes)**: China • Jiangxi Province, Mount Matoushan, Baishakeng, on rocky cliff, 27.77°N, 117.23°E, 424 m a.s.l. 21 May 2024, *Xiong Y. 240521* (SYS00236993) • Longjing, in soily slope, 27.79°N, 117.24°E, 375 m a.s.l., *Xiong Y. s.n.*, *Li E.X & Li*, *J.X. NCU2016MTS0221* (JXU0017056) • Zhejiang Province, Jiande City, Daciyan, *Dai J.M 24040701* (SYS00237017), Wenzhou City, Baizhangji, *Dai J.M*. (SYS00237018). ***Sedumemarginatum***: China • Zhejiang Province, *Migo H. s.n.* (ZNAS00332278, NAS00071019, NAS00332264). ***Sedumbaileyi***: China • Jiangxi Province, Lushan Mountain, *Peng Y.S. 21051101* (SYS). ***Sedummakinoi***: Japan • Nanokawa, Tosa, *Makino T. 93* (LE01014732, LE01014733), *Watanabe, K. s.n.* (HUH01989208) • Nagasaki, *Anonymous s.n.* (K000732545) • Okayama, *Furuse M. 52653* (PE01135685, PE01135684), *Furuse M. 51887* (PE01135678), *Furuse M. 52825* (PE01135672), *Furuse M. 52653* (PE01135686) • Yamaguchi, *Saito, S. 3173* (PE01458488), *Nikai J. s.n.* (TNS-VS-48304) • Tochigi, *Furuse M. 14896* (PE01135683), *Furuse M. 54759* (PE01135687), *Nakamura s.n.* (KAG046500), *Yoshi K. 14896* (KAG175186) • Kumamoto, *Sumihiko H. 44370* (KAG046490) • Saga, *Bajou I. s.n.* (KAG046495) • Shizuoka, *Hideaki N. 4493* (KAG046499) • Oita, *Sumihiko H. 44371* (KAG046504) • Hiroshima, *Taizo M. 165* (KAG046497) • Saitama, *Shigeki K. 1171* (KAG046502) • Hyogo, *Fukuoka N. 13638* (TNS-VS-564993) • Oity, *Yamaki N.*, *Herb. H. Koidzumi 97349* (TNS-VS-480622), *Herb. H. Koidzumi 97536* (TNS-VS-480623) • Tokushima, *Akiyama S. 20808* (TNS-VS-775554) • Ehime, *Koidzumi H. 99692* (TNS-VS-480618).

## Supplementary Material

XML Treatment for Sedum orientalichinense

## ﻿Additional information

### Author ORCIDs

Jing-Min Dai https://orcid.org/0009-0002-1090-4823

Yu Xiong https://orcid.org/0009-0003-5804-5586

Pan Li https://orcid.org/0000-0002-9407-7740

Yue-Liang Xu https://orcid.org/0000-0002-4137-4397

Qiang Fan https://orcid.org/0000-0003-4254-6936

### Data availability

All of the data that support the findings of this study are available in the main text or Supplementary Information.

## Supplementary materials

Supplementary material 1 Taxa, voucher information, GenBank accession numbers and references for ITS sequences of Sedum (S.) species and three outgroups used for phylogenetic analyses in this study Data type docx https://binary.pensoft.net/file/1281240This dataset is made available under the Open Database License (http://opendatacommons.org/licenses/odbl/1.0/). The Open Database License (ODbL) is a license agreement intended to allow users to freely share, modify, and use this Dataset while maintaining this same freedom for others, provided that the original source and author(s) are credited.Jing-Min Dai, Yu Xiong, Pan Li, Yue-Liang Xu, Qiang Fan
