# Supplementary material for: ﻿A new species of Sedum (Crassulaceae) from eastern China based on morphological and molecular evidence
Source: PhytoKeys. 2025 Mar 14;253:271–85. doi: 10.3897/phytokeys.253.119922 (PMC11929005; doi:10.3897/phytokeys.253.119922)
Supplement: Supplementary material 1 — Taxa, voucher information, GenBank accession numbers and references for ITS sequences of Sedum (S.) species and three outgroups used for phylogenetic analyses in this study [file phytokeys-253-271_article-119922__-s001.docx]

**Table S1**. Taxa, voucher information, GenBank accession numbers and references for ITS sequences of *Sedum* (*S*.) species and three outgroups used for phylogenetic analyses in this study.

| Taxon | Voucher | Accession number | Reference |
| --- | --- | --- | --- |
| *S. actinocarpum* | *Ito 1749* | LC229265 | Ito et al. 2017a |
| *S. alfredi* | *Kokubugata 17190* | AB930259 | Ito et al. 2014a |
|  | *Kokubugata 17191* | AB930260 | Ito et al. 2014a |
|  | *Kokubugata 17192* | AB930261 | Ito et al. 2014a |
|  | *WUK415208* | FJ919953 | Wang and Shu unpublished |
| *S. baileyi* | *Peng Y.S. 21051101* | PP981035 | (published here) |
| *S. bergeri* | *Ni et al.* | AY352897 | Ni et al. unpublished |
| *S. boninense* | *Ito 2371* | LC229242 | Ito et al. 2017a |
| *S. bulbiferum* | *Ito 416* | LC229234 | Ito et al. 2017a |
|  | *130514hs41* | KM111166 | Xie et al. 2014 |
|  | *130524qz09* | KM111165 | Xie et al. 2014 |
| *S. emarginatum* | *130512hs27* | KM111145 | Xie et al. 2014 |
|  | *24041001* | PP981038 | (published here) |
|  | *24052201* | PP981037 | (published here) |
| *S. erici-magnusii* | *Ito 2077* | LC229235 | Ito et al. 2017A |
| *S. erythrospermum* | *Tsutsumi 1504* | AB906473 | Ito et al. 2014b |
| *S. formosanum* | *Ito 1260* | LC229279 | Ito et al. 2017a |
| *S. hakonense* | *Mayuzumi C00005* | AB088625 | Mayuzumi and Ohba 2004 |
| *S. hangzhouense* | *Ito 2604* | LC260130 | Ito et al. 2017b |
| *S. japonicum* | *Kokubugata 16749* | AB906475 | Ito et al. 2014b |
| *S. japonicum subsp. uniflorum* | *Ito 447* | LC229241 | Ito et al. 2017a |
| *S. japonicum var. oryzifolium* | *Ito 2285* | LC229239 | Ito et al. 2017a |
| *S. japonicum var. pumilum* | *Ito 2287* | LC229240 | Ito et al. 2017a |
| *S. japonicum var. senanense* | *Ito 2200* | LC229238 | Ito et al. 2017a |
| *S. jinglanii* | *Y. S. Huang 21040301* | OP288035 | Huang et al. 2023 |
| *S. jiulungshanense* | *Ito 76* | LC229243 | Ito et al. 2017a |
| *S. kiangnanense* | *CMQ1030* | LC229244 | Ito et al. 2017a |
| *S. lineare* | *Mayuzumi C00120* | AB088623 | Mayuzumi and Ohba 2004 |
| *S. lipingense* | *ZRB1479* | MN150061 | Zhang et al. 2019 |
| *S. lungtsuanense* | *Ito 3563* | LC260131 | Ito et al. 2017b |
| *S. makinoi* | *Ito 626* | AB930280 | Ito et al. 2014 |
|  | *Ito709* | LC260132 | Ito et al. 2017a |
|  | *Kokubugata 16730* | AB906476 | Ito et al. 2014b |
|  | *Mayuzumi C00086* | AB088627 | Mayuzumi et al. 2004 |
|  | *Umemoto2325* | LC229246 | Ito et al. 2017b |
| *S. mexicanum* | *Ito 647* | LC229247 | Ito et al. 2017a |
| *S. morrisonense* | *Ito 2765* | LC229290 | Ito et al. 2017a |
| *S. multicaule* | *Miyamoto et al. TI9596136* | AB088631 | Mayuzumi and Ohba 2004 |
| *S. nagasakianum* | *Ito 2064* | LC229249 | Ito et al. 2017a |
| *S. nokoense* | *Kokubugata 10426* | AB906478 | Ito et al. 2014b |
| *S. oligospermum* | *Ito 74* | LC229250 | Ito et al. 2017a |
| *S. oreades* | *Rao 090803-03* | KF113733 | Zhang et al. 2014 |
| *S. orientalichinense* | *Xiong Y. 23062901* | PP116144 | (published here) |
|  | *Xiong Y. 23062901* | PP116145 | (published here) |
|  | *Xiong Y. 23062901* | PP116146 | (published here) |
|  | *Dai J.M. 24040302* | PP989083 | (published here) |
|  | *Dai J.M. 24040701* | PP989084 | (published here) |
| *S. polytrichoides subsp. polytrichoides* | *CMQ1057* | LC229251 | Ito et al. 2017a |
| *S. polytrichoides var. setouchiense* | *Ito 2298* | LC229253 | Ito et al. 2017a |
| *S. rupifragum* | *Ito2070* | LC229254 | Ito et al. 2017a |
| *S. sarmentosum* | *Ito 978* | LC229255 | Ito et al. 2017a |
| *S. satumense* | *Ito 2295* | LC229256 | Ito et al. 2017a |
| *S. subtile* | *Ito 2259* | LC229257 | Ito et al. 2017a |
|  | *Ito 624* | AB930277 | Ito et al. 2014a |
|  | *Shimizu 1999* | AB088622 | Mayuzumi and Ohba 2004 |
| *S. taiwanianum* | *Ito 2770* | LC229297 | Ito et al. 2017a |
| *S. tetractinum* | *Ito 3623* | LC260135 | Ito et al. 2017b |
| *S. tianmushanense* | *Ito 355* | LC229261 | Ito et al. 2017a |
| *S. tosaense* | *Kokubugata 16726* | AB906483 | Ito et al. 2014b |
| *S. triactina* | *9596091* | AB088629 | Mayuzumi and Ohba 2004 |
| *S. tricarpum* | *Ito 2269* | LC229259 | Ito et al. 2017a |
|  | *Ito2274* | LC229260 | Ito et al. 2017a |
|  | *Ito3597* | LC260134 | Ito et al. 2017c |
|  | *24032601* | PP989617 | (published here) |
| *S. trullipetalum* | *Miyamoto et al. 9420132* | AB088630 | Mayuzumi and Ohba 2004 |
| *S. truncatistigmum* | *Ito 3254* | LC229306 | Ito et al. 2017a |
| *S. yabeanum* | *Ito 396* | AB906490 | Ito et al. 2014b |
| *S. zentaro-tashiroi* | *Ohba 1998* | AB088619 | Mayuzumi and Ohba 2004 |
| outgroups |  |  |  |
| *Aeonium lancerottense* | *Mort 1518* | AY082143 | Mort et al. 2002 |
| *Aeonium viscatum* | *Mort 1432* | AY082154 | Mort et al. 2002 |
| *Greenovia aizoon* | *Mort 1425* | AY082112 | Mort et al. 2002 |
